# Supplementary material for: Bio-Distribution and Pharmacokinetics of Topically Administered γ-Cyclodextrin Based Eye Drops in Rabbits
Source: Pharmaceuticals (Basel). 2021 May 18;14(5):480. doi: 10.3390/ph14050480 (PMC8158513; doi:10.3390/ph14050480)
Supplement: Supplementary file 1 [file pharmaceuticals-14-00480-s001.zip › pharmaceuticals-1204180-supplementary.pdf]

Table S1. Percent (%) drug remaining in eye drop formulations stored for 6 months under accelerated and long-term storage conditions (n=3, Mean±S.D.).

Time (months)	% drug remaining	
	1.5% (w/v) irbesartan eye drop	0.15% (w/v) candesartan eye drop
Accelerated stability (40°C, 75%RH)		
0 Month	101.69±1.85	95.09±3.41
1 Month	99.23±2.26	87.82±4.53
3 Month	103.70±2.06	84.67±5.28
6 Month	96.35±2.94	82.83±11.55
Long-term stability (25°C, 60%RH)		
0 Month	101.69±1.85	95.09±3.41
1 Month	100.22±1.69	95.37±4.27
3 Month	102.76±1.68	87.72±5.38
6 Month	100.67±4.55	88.56±5.15
